# Fenofibrate suppresses corneal neovascularization by regulating lipid metabolism through PPARα signaling pathway

**DOI:** 10.3389/fphar.2022.1000254

**Published:** 2022-12-16

**Authors:** Tong Zhou, Ke Yan, Yuhan Zhang, Linfangzi Zhu, Yi Liao, Xiaoxiang Zheng, Yongxiong Chen, Xiaoxin Li, Zuguo Liu, Zhaoqiang Zhang

**Affiliations:** ^1^ Eye Institute and Affiliated Xiamen Eye Center of Xiamen University, School of Medicine, Xiamen University, Xiamen, Fujian, China; ^2^ Department of Pharmacy, Xiang’an Hospital of Xiamen University, School of Medicine, Xiamen University, Xiamen, China; ^3^ Fujian Provincial Key Laboratory of Ophthalmology and Visual Science, Fujian Engineering and Research Center of Eye Regenerative Medicine, Fujian Provincial Key Laboratory of Corneal and Ocular Surface Diseases, Xiamen, China; ^4^ The First Affiliated Hospital, Department of Ophthalmology, Hengyang Medical School, University of South China, Hengyang, China; ^5^ Department of Ophthalmology and Clinical Centre of Optometry, Peking University People’s Hospital, Beijing, China

**Keywords:** cornea, neovascularization, fenofibrate, PPARα, lipid metabolism, lipid deposition

## Abstract

**Purpose:** The purpose of this study was to explore the potential underlying mechanism of anti-vascular effects of peroxisome proliferator–activated receptor α (PPARα) agonist fenofibrate against corneal neovascularization (CNV) through the changes of lipid metabolism during CNV.

**Methods:** A suture-induced CNV model was established and the clinical indications were evaluated from day 1 to day 7. Treatments of vehicle and fenofibrate were performed for 5 days after suture and the CNV areas were compared among the groups. The eyeballs were collected for histological analysis, malondialdehyde (MDA) measurement, terminal deoxynucleotidyl transferase 2′-deoxyuridine 5′-triphosphate nick end labeling (TUNEL) staining, western blot, quantitative real-time PCR (qRT-PCR) assays and immunohistochemical (IHC) staining to elucidate pathological changes and the underlying mechanism.

**Results:** Lipi-Green staining and MDA measurement showed that lipid deposition and peroxidation were increased in the CNV cornea while the expression of long-chain acyl-coenzyme A synthetase 1 (ACSL1), carnitine palmitoyltransterase 1A(CPT1A) and medium-chain acyl-coenzyme A dehydrogenase (ACADM), which are key enzymes of fatty acid β-oxidation (FAO) and targeted genes of peroxisome proliferator-activated receptor alpha (PPARα) pathway, were decreased in CNV cornea. Fenofibrate suppressed lipid accumulation and peroxidation damage in the CNV cornea. Fenofibrate upregulated the expression levels of PPARα, ACSL1, CPT1A, and ACADM compared with vehicle group. IHC staining indicated that fenofibrate also decreased the expression of VEGFa, VEGFc, TNFα, IL1β and CD68.

**Conclusion:** Disorder of lipid metabolism may be involved in the formation of suture-induced CNV and fenofibrate played anti-neovascularization and anti-inflammatory roles on cornea by regulating the key enzymes of lipid metabolism and ameliorating lipid peroxidation damage of cornea through PPARα signaling pathway.

## 1 Introduction

Corneal neovascularization (CNV) can resulte from various etiologies, including corneal infections, contact lens wearing, ocular surface inflammation, corneal chemical injury and injury including limbal stem cell deficiency (LSCD) (Lee et al., 1998; Hamill et al., 2013; Roshandel et al., 2018). CNV can eventually lead to loss of corneal transparency and decreased visual acuity (Wang et al., 2019). CNV is a main hazard factor associated with corneal graft rejection and subsequent treatment failure (Bachmann et al., 2010). Multiple mechanisms related with the incidence of CNV were confirmed including inflammation, limbal stem cell deficiency, oxidative stress as well as the imbalance between angiopoietin and anti-angiogenesis factors (Peng et al., 2022). However, anti-vascular endothelial growth factor (VEGF) and anti-inflammatory pharmaceuticals remain the mainstay of therapy for CNV up to now while there are risks of significant adverse effects (steroid-induced glaucoma; CsA-induced eczema, chest pain, back pain) with these treatments (Roshandel et al., 2018; Zhang et al., 2022). Anti-VEGF therapy is the main FDA approved anti-angiogenic treatment for a range of solid tumors (Donovan et al., 2019). The introduction of VEGF-targeting therapies in ophthalmology has revolutionized the treatment of neovascular (wet/exudative) age-related macular degeneration (nAMD), retinal vein occlusion and diabetic macular edema (Pan et al., 2020). However, they are not always effective in all of the patients. VEGF is one of the critical mediators of corneal neovascularization but current anti-VEGF therapies have produced limited results in the cornea because of their short half-lives (Feizi et al., 2017; Than et al., 2018; Su et al., 2021) and side effects, such as cornea scar. And the need for an FDA-approved potent topical antiangiogenic agent is still unmet. The advent of newer more potent and effective competitive anti-angiogenic agents is urgently needed for better therapy.

It is well known that disorders of lipid metabolism and lipid deposition are associated with ocular diseases (Barchiesi et al., 1991). Lipid metabolism was reported to be related with vascular growth and the course of pathologic neovascularization (Sapieha et al., 2011; Yanai et al., 2014). Fatty acid β-oxidation (FAO), an important process in lipid metabolism, works depending on several enzymes such as long-chain acyl-CoA synthetase (ACSL), carnitine palmitoyltransterase (CPT) and acyl-CoA dehydrogenase (ACAD). ACSL1, one of the ACSL family members found in the cytoplasm, mainly directs fatty acids toward β-oxidation by converting long-chain free fatty acids to acyl-CoA esters (Ellis et al., 2010). CPT, the rate-limiting enzyme in the carnitine cycle, is essential for the transmembrane transport of long-chain acyl-CoAs to the mitochondrial matrix in the subsequent step. Once inside the mitochondrial matrix, the long-chain acyl-CoAs enter the β-oxidation spiral and ACAD is an important enzyme in the following oxidation steps (Houten and Wanders, 2010; Ruiz-Sala and Peña-Quintana, 2021). Our previous study found the expression level of CPT1A, a member of CPT family, was decreased in sleep-deprived mouse corneas (Tang et al., 2018). Moreover, CPT1A was reported to be responsible for the angiogenesis diseases (Schlaepfer and Joshi, 2020). However, few research focused on the changes of other enzymes in FAO such as ACSL and ACAD in angiogenesis.

Peroxisome proliferator-activated receptor alpha (PPARα), one of PPAR family members, is a nuclear receptor which regulates the expression of various target genes to affect lipid metabolism, inflammatory response and so on (Bougarne et al., 2018). Increasing evidences suggested that PPARα modulates angiogenesis in conditions including tumor growth and ocular neovascularization (Tomita et al., 2020; Wagner and Wagner, 2020). PPARα is an important therapeutic target for various types of diseases in clinics, for example, dyslipidemia, cardiovascular diseases, and diabetes. A well-known PPARα agonist, fenofibrate is FDA-approved drug widely used for the treatment of hyperlipidemia with minimal side effects (Panigrahy et al., 2008) and cost-effective, having the potential to reduce the economic burden of patients. The beneficial effect of fenofibrate on the progression of diabetic retinopathy during the trial may be clinically important (Cheung and Wong, 2008; Chew et al., 2010; Hu et al., 2013; Frank, 2022). It was also demonstrated to inhibit the tube formation in human umbilical vein endothelial cells (HUVSCs) (Zhao et al., 2015) and have therapeutic effects on ocular neovascularization in animal models (Chen et al., 2013; Qiu et al., 2017). It was proposed that the beneficial effects of fenofibrate on fatty liver and cardiorenal syndrome were correlated with the enhanced gene expression of enzymes involved in β-oxidation (i.e., ASCL1, CPT1A and ACADM), which consequently prevents oxidative stress, lipid accumulation, and inflammation (Durgan et al., 2006; Harano et al., 2006; Castiglioni et al., 2018). Previously, we demonstrated that keratocytes promoted CNV through VEGFr3 induced by PPARα-inhibition (Wang et al., 2020). However, PPARs may affect angiogenesis through several other factors besides VEGF. It may regulate intracellular lipid metabolic pathways to cause microvascular formation (Hiukka et al., 2010; Gong et al., 2016b; Yu et al., 2016). It is still unclear whether PPARα plays a role on CNV in a lipid-related way, and the underlying mechanisms require further explorations.

In this study, we confirmed the anti-neovascular effects of fenofibrate in the CNV model induced by corneal suture. Corneal alkali burn and corneal suture are commonly used to induce corneal neovascularization. As previously mentioned, CNV model induced by suture in animal models displayed better practical advantages (Shi et al., 2010; Giacomini et al., 2014). Thus, we used the CNV model induced by suturation in our present study, which was a more dominant and specific model to investigate the pathogenesis and treatment of CNV. We elucidated the possible mechanisms focusing on the roles of key enzymes of FAO, which are downstream target genes of PPARα pathway, in lipid metabolism in animal model. It was a continuous work of our previous studies from our group (Ma et al., 2014; Zhou et al., 2014; Tang et al., 2018; Wang et al., 2020) to provide a fresh perspective and better understandings of the etiology of CNV and supply new evidences for its novel potential therapies.

## 2 Materials and methods

### 2.1 Reagents and antibodies

Anti-PPARα (ab8934), anti-CPT1A (ab220789), anti-CD146 (ab5769) anti-TNFα (ab66579) primary antibodies were obtained from Abcam Plc. (Cambridge, England). Anti-IL1β (A16288) and anti-CD68 (A6554) primary antibodies were obtained from ABclonal Technology Co., Ltd. (Wuhan, Hubei, China). Anti-VEGFa (19003-1-AP), anti-VEGFc (22601-1-AP), Anti-ACSL1 (13989-1-AP), anti-ACADM (55210-1-AP) and anti-ALOX5 (10021-1-Ig) primary antibodies were obtained from Proteintech Inc. (Wuhan, Hubei, China). Anti-β-actin-peroxidase antibody (A3854), goat anti-rabbit IgG (whole molecule)-peroxidase antibody (A0545), bisBenzimide trihydrochloride (Hoechst33342) (14533) and dimethyl sulfoxide (DMSO) (276855) were obtained from Sigma-Aldrich Corp (St. Louis, MO, United States). N-Histofine simple stain MAX PO kits (anti-rabbit) (414142F) were purchased from Nichirei Biosciences (Tokyo, Japan); Lipi-Green reagent (LD02) was obtained from Dojindo Laboratorise (Kumamoto, CA, Japan). Fenofibrate (ab120832) was obtained from Abcam Plc. (Cambridge, England). Liquid DAB + substrate chromogen system kit (K3468) was purchased from Dako Agilent Technologies (Santa Clara, CA, United States).

### 2.2 Mice corneal suture model and treatment

The C57BL/6 mice (male, aged 6–8 weeks) were purchased from Beijing Vital River Laboratory Animal Technology Co., Ltd. (Beijing, China). All animals were kept in standard environment as follows: relative humidity 60% ± 10%, room temperature 25°C ± 1°C, 12 h light-dark cycles, food and water *ad libitum*. All the animal experiments were implemented in this study according to the Association for Research in Vision and Ophthalmology (ARVO) Statement for the Use of Animals in Ophthalmic and Vision Research, and the experimental scheme was approved by the laboratory animal ethics committee of Xiamen University (ID Number: XMULAC 20180053).

The mouse CNV model was induced by cornea suture. The operation of mice was carried out under a general anaesthetic by intraperitoneal injection of pentobarbital (40–50 mg/kg). One to two drops of 0.5% proparacaine ophthalmic solution was topically applied to added to mice ocular surface. Three 10–0 nylon sutures were conducted in the cornea between corneal center and the limbus at the 12, 4, and 8 o’clock position, respectively. The mice were put on heating pad softly and closely monitored after the surgery, returned to cages after waking up, fed a standard rodent diet and observed daily. Experimental mice were sacrificed at different time points (D1, D3, D5 and D7) after suture. The normal control mice corneas were taken at D0.

In this study, mice corneas were topically applied with 200 μM fenofibrate (dissolved in DMSO and further diluted with saline) for five consecutive days after suture, while control group mice were applied with vehicle (saline containing DMSO, same volume as 200 μM fenofibrate, 0.1% DMSO) (5 μl each time, 3 times per day) as previously published (Wang et al., 2020). Experimental mice were sacrificed at D5 after suture.

After Experimental mice were sacrificed at different time points, the eyeballs and periocular adnexa of mice were separated and collected, and then embedded in Tissue-Tek O. C. T. compound (Sakura Finetek Inc., Torrance, CA, United States), or fixed in 4% paraformaldehyde 12 h and then embedded in paraffin following standard protocol, the corneas were collected for qRT-PCR or western blot.

### 2.3 Evaluation of corneal neovascularization

Images of mice cornea were captured by a slit-lamp microscope (Takagi Seiko Co., Ltd., Nagano, Japan), and the new blood vessels were quantified as previously described (Zhou et al., 2014). As described briefly below, four quadrants were divided from the cornea and the vessel length of each quarter (Li, i = 1–4) was measured. The CNV area (A) was calculated using the below equation: A = ∑_i=1−4_ 3.1416 × [*R*_−_^2^(R_−_Li)^2^]. (R = 2 mm, the radius of mouse cornea).

### 2.4 Histologic staining and immunostaining

Hematoxylin and eosin (H&E) staining was implemented on the paraffin sections following standard protocol. Sections (5 μm) were deparaffinized and stained with haematoxylin, differentiated with 1% acid alcohol. After eosin staining, the sections were dehydrated, hyalinized-and mounted with H-5000 mounting medium (Vector Laboratories, Inc., Burlingame, CA, United States). Optics electronic microscope (Eclipse 50i, Nikon, Japan) was performed to observe and examine the images of samples slides.

Paraffin sections or frozen sections were used for immunohistochemical (IHC) staining according to standard protocols. For paraffin sections, antigen unmasking was required using Tris-EDTA buffer (pH 9.0). The tissue endogenous peroxides activity was quenched with blocking solution (3% hydrogen peroxide). The slides were incubated with 2% bovine serum albumin (BSA). After tipped off excess serum from cextion, the primary antibodies of TNFα (1:100), IL1β (1:200), CD68 (1:200), VEGFa (1:300), VEGFc (1:300), PPARα (1:200), CPT1A (1:400), ACSL1 (1:300) and ACADM (1:300) were applied and incubated at 4°C overnight (8–12 h). After washed in 1 × PBS buffer, the sections were incubated with N-Histofine simple stain MAX PO kit, developed with Liquid DAB + substrate chromogen system. Then sections were mounted with H-5000 mounting medium, observed and examined with an optics electronic microscope.

### 2.5 Lipi-Green staining

A lipid droplet-specific fluorescent probe for tissue frozen slides was used for lipid staining according to the manufacturer’s protocol. After incubated at 37°C for 1 h or 4°C overnight with the Lipi-Green probe,the sections were washed for 5 min in 1 × PBS buffer 3 times and counterstained with Hoechst33342, mounted with H-1000 mounting medium (Vector Laboratories, Inc., Burlingame, CA, United States) and observed on a Zeiss LSM 880 + Airyscan confocal laser microscope (Zeiss, Oberkochen, Germany).

### 2.6 TUNEL staining

The DeadEnd™ Fluorometric TUNEL System (G3250, Promega, Madison, WI) was used to measures nuclear DNA fragmentation, an important biochemical hallmark of apoptosis in many cell types. Paraffin-embedded sections were performed to assay apoptotic cell death according to the manufacturer’s standard protocol. After the nuclear counterstain with Hoechst 33342, slides were mounted H-1000 mounting medium and observed on a Zeiss LSM 880 + Airyscan confocal laser microscope (Zeiss, Oberkochen, Germany).

### 2.7 MDA detection

Malondialdehyde (MDA) detection kit (A003-2, Nanjing Jiancheng Bioengineering Institute, Nanjing, China) was employed to detecte corneal MDA content according to the manufacturer’s protocol. Absorbance was measured spectrophoto metrically at 586 nm. The MDA content of cornea was quantified by the following formula: MDA content (nmol/mg protein) = [(sample absorption−sample blank absorption)/(standard substance absorption−standard substance blank absorption)] × 10 nmol/protein content of sample.

### 2.8 Western blot assay

Mice corneal proteins were extracted with ice-precooling RIPA buffer (89901; Thermo Fisher Scientific, Waltham, MA, United States) containing protease inhibitor cocktail (78440; Thermo Fisher Scientific). Pierce™ BCA Protein Assay Kit (23227; Thermo Fisher Scientific) was used for the quantitation of total protein solutions. For immunodetection of proteins, the protein samples were denatured by heating at 100°C for 10 min. After electrophoresis transfer, proteins were subjected and separated from SDS-PAGE Gels to PVDF membrane followed by incubating with blocking solution (2% BSA) for 30 min to reduce non-specific bindings. Then the membranes were incubated with primary antibodies of PPARα (1:1,000), CPT1α (1:8,000), ACSL1 (1:2,000), ACADM (1:2,000), ALOX5 (1:2000), and β-actin (1:10,000) at 4°C for 8–12 h or overnight. After washing with Tris-buffer saline (0.1% Tween-20) for 10 min 3 times, the PVDF membranes were incubated with secondary antibody, HRP-conjugated goat anti-rabbit IgG (1:10,000) or goat anti-mouse IgG (1:10,000) at room temperature for 1 h. SuperSignal West Femto Chemiluminescent Substrate (34095, Thermo Fisher Scientific, Waltham, MA, United States) was used for detection. The blots were imaged using ChemiDox XRS system (BioRad Laboratories, Inc., Philadelphia, PA, United States) at the indicated times. Quantitation of signal intensity was performed using ChemiDox XRS system software.

### 2.9 RNA isolation and quantitative real time PCR

Total RNA was extracted from cornea using TRIzol reagent (15596026, Thermo Fisher Scientific) according to standard methods. The first strand cDNA was synthesized by using RevertAid First Strand cDNA Synthesis Kit (K1622, Thermo Fisher Scientific) according to the manufacturer’s protocol. Quantitative PCR (qRT-PCR) was performed to determine the mRNA expression of *Pparα*, *Cpt1a*, *Acsl1*, *Acadm*, *Alox5* and *Actin* using TaKaRa TB Green^®^ Premix Ex Taq (Tli RNaseH Plus) (RR420A, Kusatsu, Shiga, Japan) in Step OnePlus Real-Time PCR System (Thermo Fisher Scientific) in accordance with the product manual. All reactions were performed in triplicate and the delta comparative threshold cycle (CT) method was used for data analysis. All data were normalized to *Actin* expression and expressed as flod changes of the control group. The primers used in this experiment are described as Table 1.

**TABLE 1:** The mouse primers used in this experiment.

Gene	Forward primer (5′-3′)	Reverse primer (5′-3′)
*Acsl1*	AGT​CAG​TGG​AAA​TAG​CGG​GTA	CCT​CTG​GAA​GCC​ATC​GTA​CA
*Acadm*	GGA​GTA​CCC​GTT​CCC​TCT​CA	GCA​CCC​CTG​TAC​ACC​CAT​AC
*Alox5*	CCCACGGGGACTACATCG	ACA​GGT​TCT​CCA​TCG​CTT​TTG​A
*Cpt1a*	AAC​CCA​TAT​TCA​GGC​AGC​GA	GAC​GTG​TTG​GAT​GGT​GTC​TGT
*Pparα*	GTC​TGT​CGG​GAT​GTC​ACA​CAA	TCA​TGT​ATG​ACA​AAA​GGC​GGG
*Actin*	AGA​TCA​AGA​TCA​TTG​CTC​CTC​CT	ACG​CAG​CTC​AGT​AAC​AGT​CC

### 2.10 Image analysis

To analyze the positive immunostaining ratio of cells, images of Lipi-Green staining, TUNEL staining and IHC staining were processed by ImageJ software version 6 (NIH, Bethesda, MD). The optical density [integrated optical density (IOD)] and area of all the collected images were measured using the ImageJ image analysis system (NIH), and the average optical density [mean density (MD)] of each image was calculated. Each sample was analyzed in at least three different areas.

### 2.11 Statistical analysis

The data analysis of CNV area, detection of qRT-PCR and MDA were performed with GraphPad Prism 8.0.1 software. The values are presented as mean ± standard error of mean (SEM). Data analysis was performed with a one-way analysis of variance (ANOVA) with Tukey HSD *post hoc* tests. *p* ≤ 0.05 was considered statistically significant, n = 3 to 6 in each group.

## 3 Results

### 3.1 Lipid deposition and inflammation in a CNV model established by suture

**FIGURE 1:**
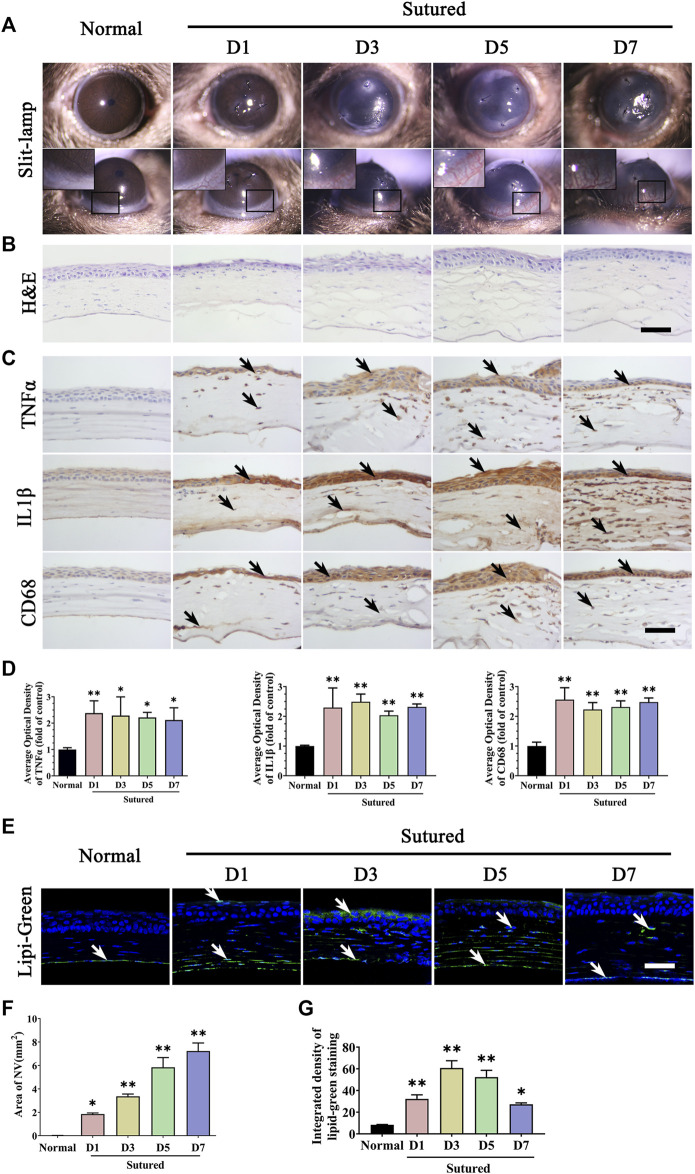
Lipid deposition and inflammation in corneal neovascularization model established by suture. **(A)** Clinical observation of corneal neovascularization using slit-lamp. From left to right are normal cornea without suturation or any treatment, sutured cornea on day 1 to day 7 respectively. **(B)** H&E staining of central cornea in normal cornea and sutured cornea on day1 to day7. Scale bar: 50 μm. **(C)** Images of IHC staining of cornea paraffin sections stained with antibody of TNFα, IL1β and CD68 in normal cornea and sutured cornea on day 1 to day 7 (IHC staining is labeled brown and indicated by black arrows), Scale bar: 50 μm. **(D)** Statistic analysis of images of IHC staining with TNFα, IL1β, CD68 between normal cornea and sutured cornea on day 1 to day 7 separately, n = 4 in each group. **(E)** Lipi-Green staining (green labeling, which is indicated by white arrows) of normal and sutured cornea on day 1 to day 7 severally. Scale bar: 50 μm. **(F)** The statistic analysis of NV area in normal cornea and sutured cornea on day 1 to day 7, n = 3 in each group. (**G)** Statistic analysis of Lipi-Green staining in normal cornea and sutured cornea on day 1 to day 7, n = 3 in each group. **indicates *p* < 0.01 and *indicates *p* < 0.05, one-way analysis of variance (ANOVA). All Data are shown as mean ± SEM.

To observe lipid deposition in CNV, we selected and applied mouse suture-induced CNV model and clinical indication of CNV was examined on day 1, day 3, day 5 and day 7. It was observed that new vessels invaded from limbal region after suturation and grew longer and denser from day 1 to day 7 (Figure 1A). The NV area calculation showed a gradually and significantly increase from day 1 to day 7 compared with normal group (Figure 1F, *p* < 0.05). We also investigated the morphological change of cornea tissue through H & E staining and detected corneal inflammation by IHC staining with TNFα, IL1β, and CD68. The H & E staining results showed the structural alterations of corneal epithelium and inflammatory cell infiltration and edema in the corneal stroma after suturation (Figure 1B), and IHC staining suggesting a significantly increased inflammation during neovascularization compared to normal cornea (Figures 1C, D, *p* < 0.05). We further detected the lipid deposition in the cornea by Lipi-Green staining (Figures 1E, G, *p* < 0.05). The results showed a statistically significant increase of lipid accumulation in suture cornea both in corneal epithelium and stroma on day 1 to day 7 compared with normal group. These results indicated the formation of CNV was accompanied with lipid deposition after corneal suture.

### 3.2 Lipid peroxidation damage of cornea in suture-induced CNV

**FIGURE 2:**
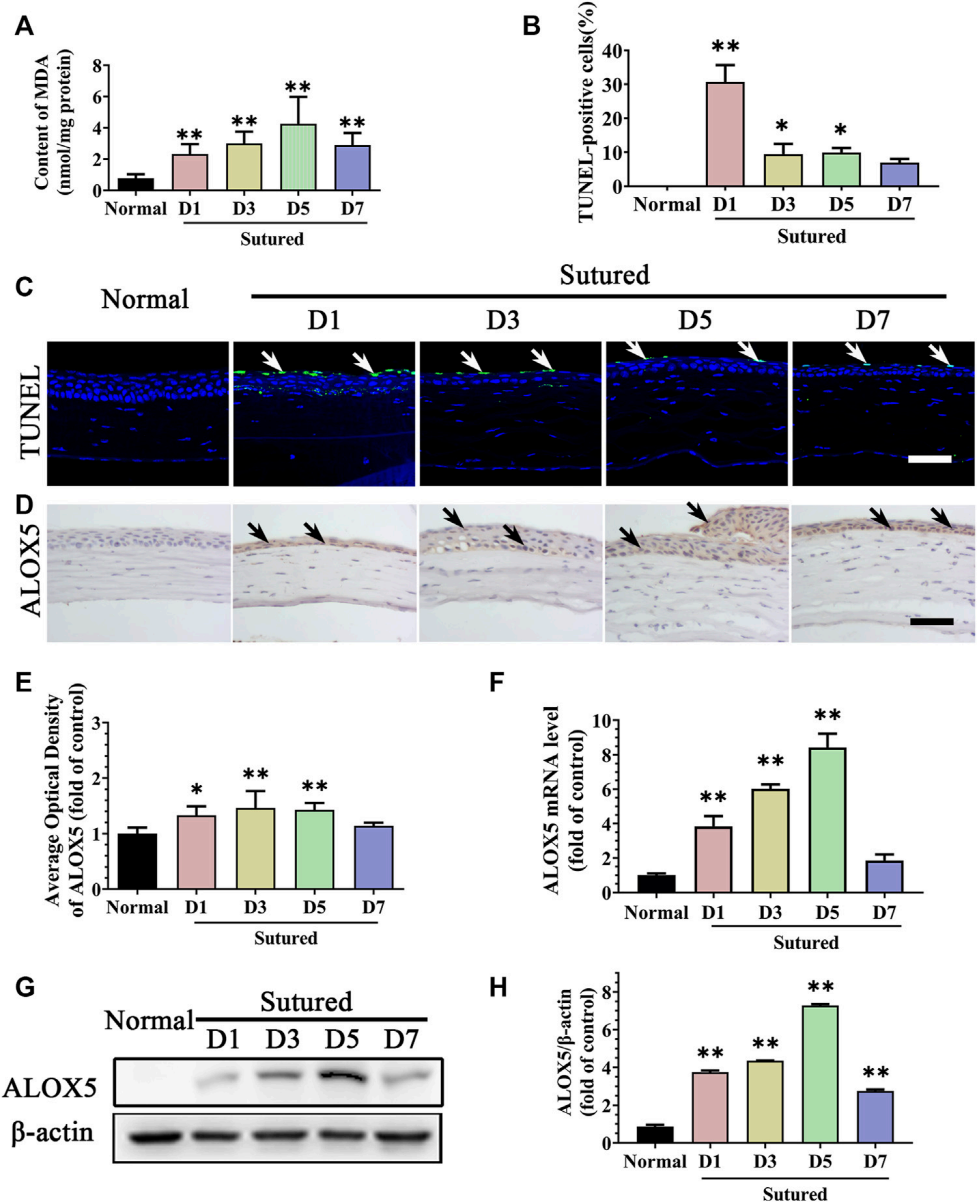
Lipid peroxidation damage of cornea in suture-induced CNV. **(A)** The statistic comparation of the data of MDA content between normal cornea and sutured cornea on day 1 to day 7 severally, n = 6 in each group. **(C,B)** Images of TUNEL staining (green labeling, which is indicated by white arrows) of central cornea in normal cornea and sutured cornea on day 1 to day 7 severally and statistic analysis of TUNEL staining between normal cornea and sutured cornea on day 1 to day 7 separately. Scale bar: 50 μm, n = 4 in each group. **(D,E)** Images and statistic analysis of IHC staining (brown labeling, which indicated by black arrows) of cornea sections stained with antibody of ALOX5 in normal cornea and sutured cornea on day 1 to day 7. Scale bar: 50 μm n = 4 in each group. **(F)** Statistic analysis of qRT-PCR assay of gene expression of *Alox5* in normal cornea and sutured cornea on day 1 to day 7, n = 3 in each group. **(G,H)** Images and analysis of western blot with antibody of ALOX5 in normal cornea and sutured cornea on day 1 to day 7, n = 5 in each group. **indicates *p* < 0.01 and *indicates *p* < 0.05, one-way analysis of variance (ANOVA). All Data are shown as mean ± SEM.

Then, lipid peroxidation in the CNV model was detected by MDA measurement. The MDA content was obviously increased in the corneal suture model compared with normal corneas (Figure 2A, *p* < 0.01). Meanwhile, the TUNEL staining showed corneal epithelium apoptosis increased observably after suture particularly on day 1 (Figures 2B,C, *p* < 0.05). All these results revealed that suturation in the cornea induced lipid peroxidation damage and subsequent apoptosis. We further examined ALOX5, an important enzyme in lipid peroxidation, whose mRNA levels and protein expression were increased in the suture model from day 1 to day 7 compared with normal cornea by IHC staining (Figures 2D,E, *p* < 0.05), qRT-PCR (Figure 2F, *p* < 0.01) and Western blot (Figures 2G,H, *p* < 0.01). Thus, the lipid peroxidation damage in the cornea during CNV may be caused by the increase of ALOX5.

### 3.3 Changes of lipid metabolism enzymes in suture-induced CNV

**FIGURE 3:**
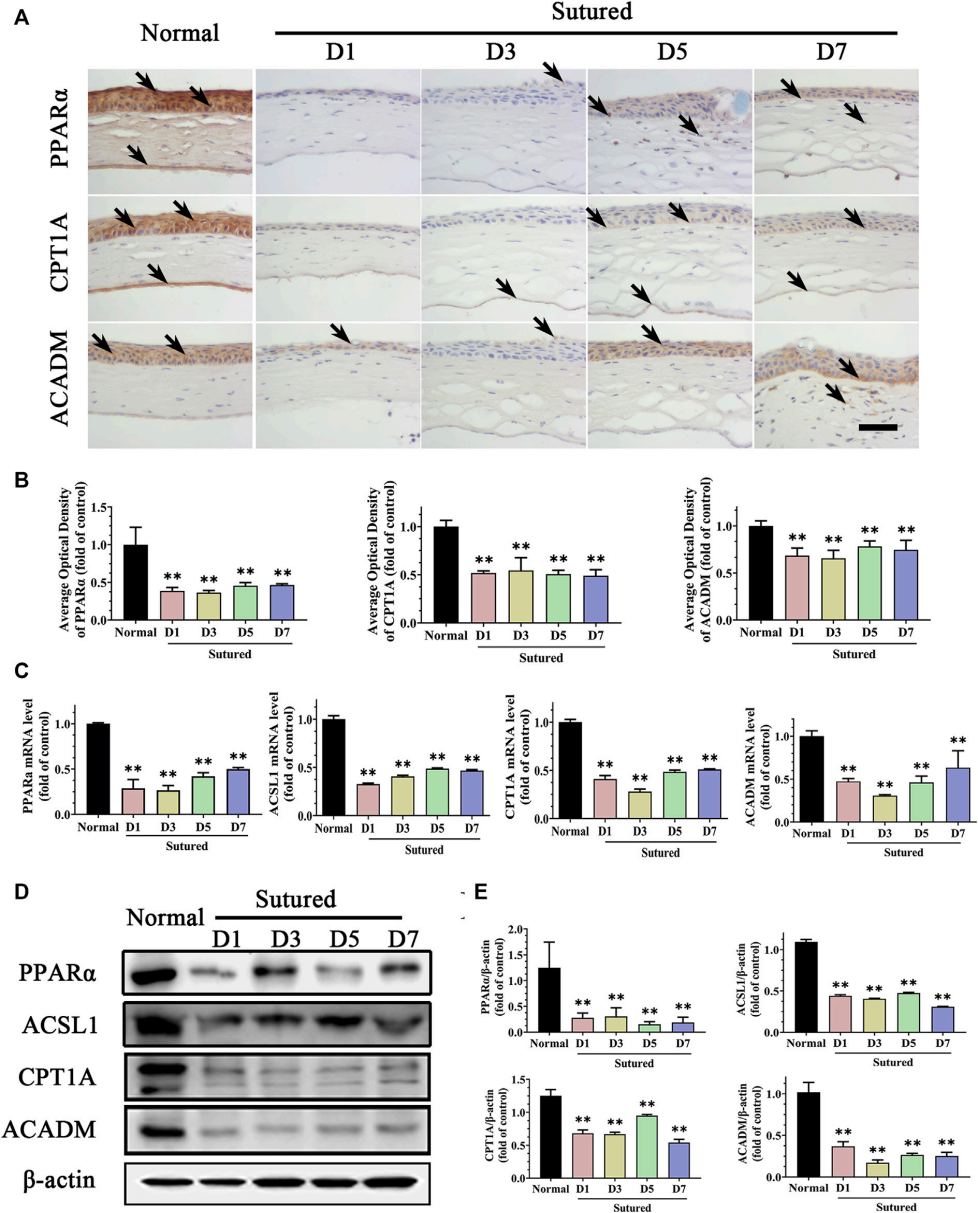
Disorder of lipid metabolism enzymes in suture-induced CNV. **(A,B)** Images and analysis of IHC staining of cornea paraffin sections with antibody of PPARα, CPT1A and ACADM in normal cornea and sutured cornea on day 1 to day 7 severally (brown labeling, which indicated by black arrows). Scale bar: 50 μm, n = 4 in each group. **(C)** Statistic analysis of qRT-PCR assay of gene expression of *Pparα*, *Ascl1*, *Cpt1a* and *Acadm*, n = 3 in each group. **(D,E)** Images of and statistic analysis of western blot with antibody of PPARα, ASCL1, CPT1A and ACADM in normal cornea and sutured cornea on day 1 to day 7, n = 4 in each group. ** denotes *p* < 0.01, one-way analysis of variance (ANOVA). Data are presented as mean ± SEM.

We further measured the PPARα pathway, which is known to be a critical pathway modulating lipid metabolism, focusing on the downstream targets including ACSL1, CPT1A, and ACADM. In the IHC staining, we found that the expressions of PPARα, CPT1A, and ACADM were located in corneal epithelium, stroma, and endothelium. The expression of PPARα was markedly reduced in the CNV cornea *via* IHC staining (Figures 3A,B, *p* < 0.01), qRT-PCR (Figure 3C, *p* < 0.01) and western blot (Figures 3D,E, *p* < 0.01). The expression levels of ACSL1, CPT1A and ACADM were decreased significantly in suture groups compared with normal group by IHC staining and analysis (Figures 3A,B, *p* < 0.01), qRT-PCR (Figure 3C, *p* < 0.01) and western blot (Figures 3D,E, *p* < 0.01). These results demonstrated downregulations of PPARα signaling pathway and its target factors which impacted the lipid metabolism during CNV pathological process. It was indicated that changes of lipid metabolism enzymes in cornea might take part in the etiologies of CNV.

### 3.4 Anti-neovascularization and lipid deposition reduction effects of fenofibrate after corneal suture

**FIGURE 4:**
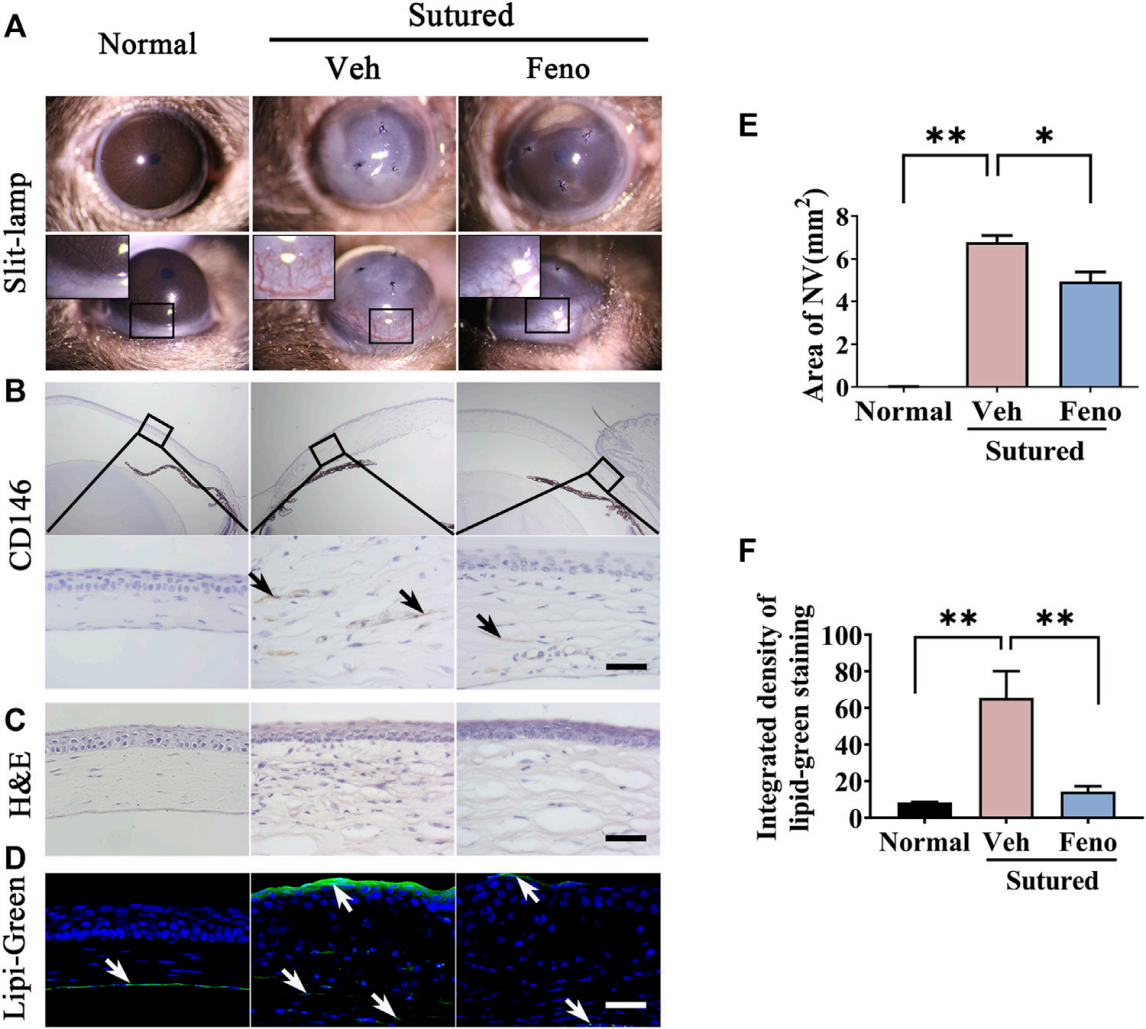
Anti-neovascularization effects of fenofibrate in suture-induced CNV. **(A–E)** Clinical observation of corneal neovascularization using slit-lamp and statistic analysis of the NV area between groups. The mice were divided into three groups: normal mouse without suturation or any treatment; sutured mouse with treatment of vehicle for 5 days; sutured mouse with treatment of fenofibrate for 5 days. (Veh, vehicle; Feno, fenofibrate, the same as below), n = 3 in each group. **(B)** Representative images of cornea sections immunostained with antibody of CD146 (IHC staining is labeled brown and indicated by black arrows). The upper and bottom rows represented images of 5 × zoom and 40 × zoom respectively. The groups were the same as in **(A)**. Scale bar: 50 μm. **(C)** H&E staining of central cornea in mouse cornea. The groups were the same as before. Scale bar: 50 μm. **(D,F)** Images of Lipi-Green staining (green labeling, which is indicated by white arrows) of mouse cornea in each group and statistic analysis of Lipi-Green staining between groups. The groups were the same as before. Scale bar: 50 μm, n = 3 in each group. ** denotes *p* < 0.01 and * denotes *p* < 0.05, one-way analysis of variance (ANOVA). Data are expressed as mean ± SEM.

We investigated the role of fenofibrate, a classical PPARα agonist, in the CNV induced by suture. The images of slit-lamp and quantification of neovascularization areas revealed that fenofibrate remarkably reduced the CNV on day 5 compared with the vehicle group (Figures 4A–E, *p* < 0.05). The IHC staining with anti-CD146 antibody further verified less vessel growth in the stroma in fenofibrate treated group. (Figure 4B). The H&E staining demonstrated fenofibrate decreased inflammatory cell infiltration and attenuated the cornea edema induced by suturation (Figure 4C). In addition, the Lipi-Green staining showed that fenofibrate significantly reduced the lipid deposition induced by suturation (Figures 4D,F, *p* < 0.01). All the results indicated anti-neovascularization,and lipid accumulation reduction effects of fenofibrate in CNV.

### 3.5 Anti-inflammation and anti-VEGF effects of fenofibrate in CNV mice model

**FIGURE 5:**
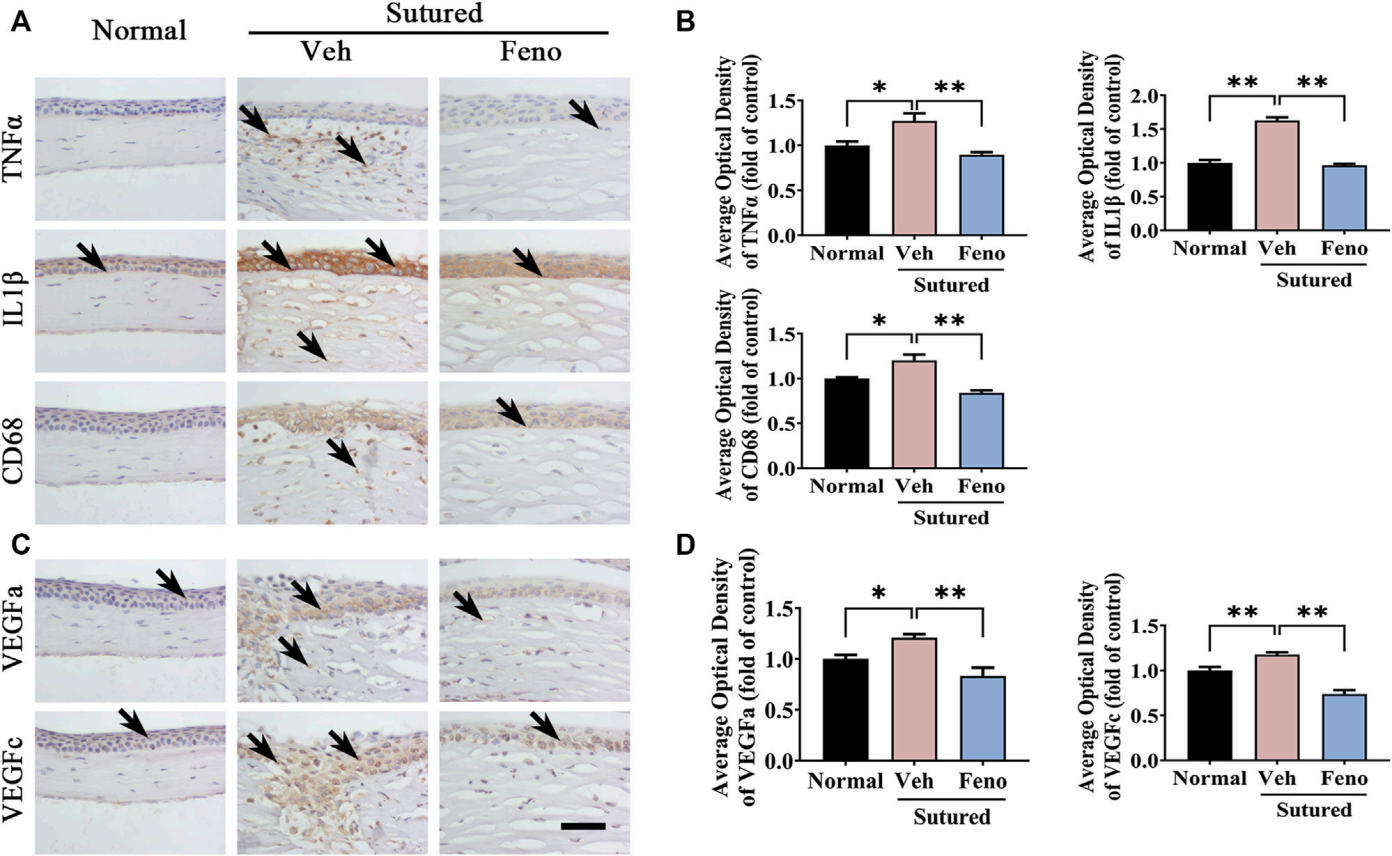
The anti-inflammatory and anti-VEGF effects of fenofibrate in suture-induced CNV. **(A)** Images of IHC staining of cornea paraffin sections stained with antibody of TNFα, IL1β and CD68 in normal cornea and sutured cornea with vehicle or fenofibrate treatment (brown labeling, which indicated by black arrows). Scale bar: 50 μm. **(B)** Statistic analysis of IHC staining with TNFα, IL1β and CD68 between groups, n = 4 in each group. **(C)** Images of IHC staining of cornea sections stained with antibody of VEGFa and VEGFc in normal cornea and sutured cornea with vehicle or fenofibrate treatment (brown labeling, which indicated by black arrows). **(D)** Statistic analysis of IHC staining with VEGFa and VEGFc between groups. Scale bar: 50 μm, n = 4 in each group. **indicates *p* < 0.01 compared to normal group, *indicates *p* < 0.05, one-way analysis of variance (ANOVA). All Data are shown as mean ± SEM.

We also observed the changes of the corneal inflammation and expression level of VEGF, which were considered to be important factors in formation of CNV. It was found that fenofibrate evidently downregulated the expression levels of TNFα, IL1β and CD68 induced by suture in IHC staining (Figures 5A,B, *p* < 0.05), which indicating the anti-inflammation effect of fenofibrate in suture mice. Simultaneously, the levels of VEGFa and VEGFc, which were critical angiogenesis factors, reduced by fenofibrate treatment after suture obviously (Figures 5C,D, *p* < 0.05). It revealed fenofibrate played anti-neovascularization role *via* both anti-inflammation and anti-VEGF effects.

### 3.6 Fenofibrate ameliorated lipid peroxidation damage in CNV

**FIGURE 6:**
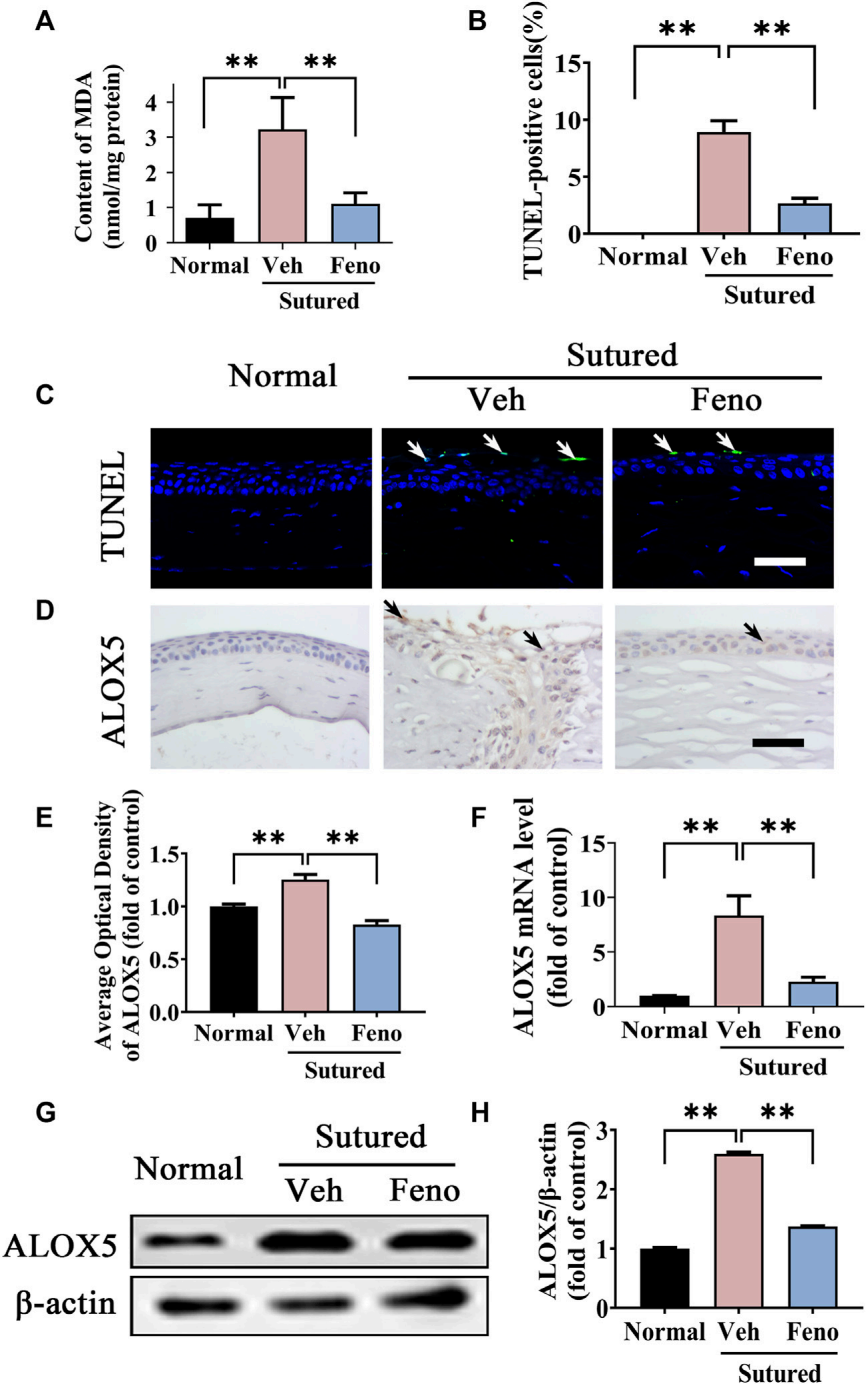
Fenofibrate ameliorated lipid peroxidation damage in CNV. **(A)** The statistic comparation of the data of MDA content between groups, n = 6 in each group. (**B,C**) Images and analysis of TUNEL staining (green labeling, which is indicated by white arrows) of central cornea in normal cornea, sutured cornea with vehicle treatment and sutured cornea with fenofibrate treatment. Scale bar: 50 μm, n = 4 in each group. **(D,E)** Images and analysis of IHC staining (brown labeling, which indicated by black arrows) with ALOX5 antibody in normal cornea, sutured cornea with vehicle treatment and sutured cornea with fenofibrate treatment. Scale bar: 50 μm, n = 4 in each group. **(F)** Statistic analysis of qRT-PCR assay of mRNA expression of *Alox5* between groups, n = 3 in each group. **(G,H)** Images of western blot with antibody of ALOX5 in normal cornea and sutured cornea with vehicle or fenofibrate treatment and statistic analysis between groups, n = 4 in each group. ** denotes *p* < 0.01, * denotes *p* < 0.05, one-way analysis of variance (ANOVA). Data are presented as mean ± SEM.

We next investigated the impact of fenofibrate on lipid peroxidation in suture-induced CNV. The MDA measurement showed that there were more lipid peroxidation products in vehicle group while fenofibrate decreased lipid peroxidation induced by suturation (Figure 6A, *p* < 0.01). The images and analysis of TUNEL staining showed fenofibrate significantly ameliorated apoptosis compared with vehicle group (Figures 6B, C, *p* < 0.01). Meanwhile, the IHC staining (Figures 6D,E, *p* < 0.01), qRT-PCR (Figure 6F, *p* < 0.01) and western blot (Figures 6G,H, *p* < 0.01) results showed the expression of ALOX5 was increased after suture and reduced in fenofibrate treatment group obviously. The results revealed a protective function of fenofibrate on the lipid peroxidation damage induced by suture by suppressing the expression of ALOX5.

### 3.7 Fenofibrate upregulated the expression levels of lipid metabolism related enzymes in CNV

**FIGURE 7:**
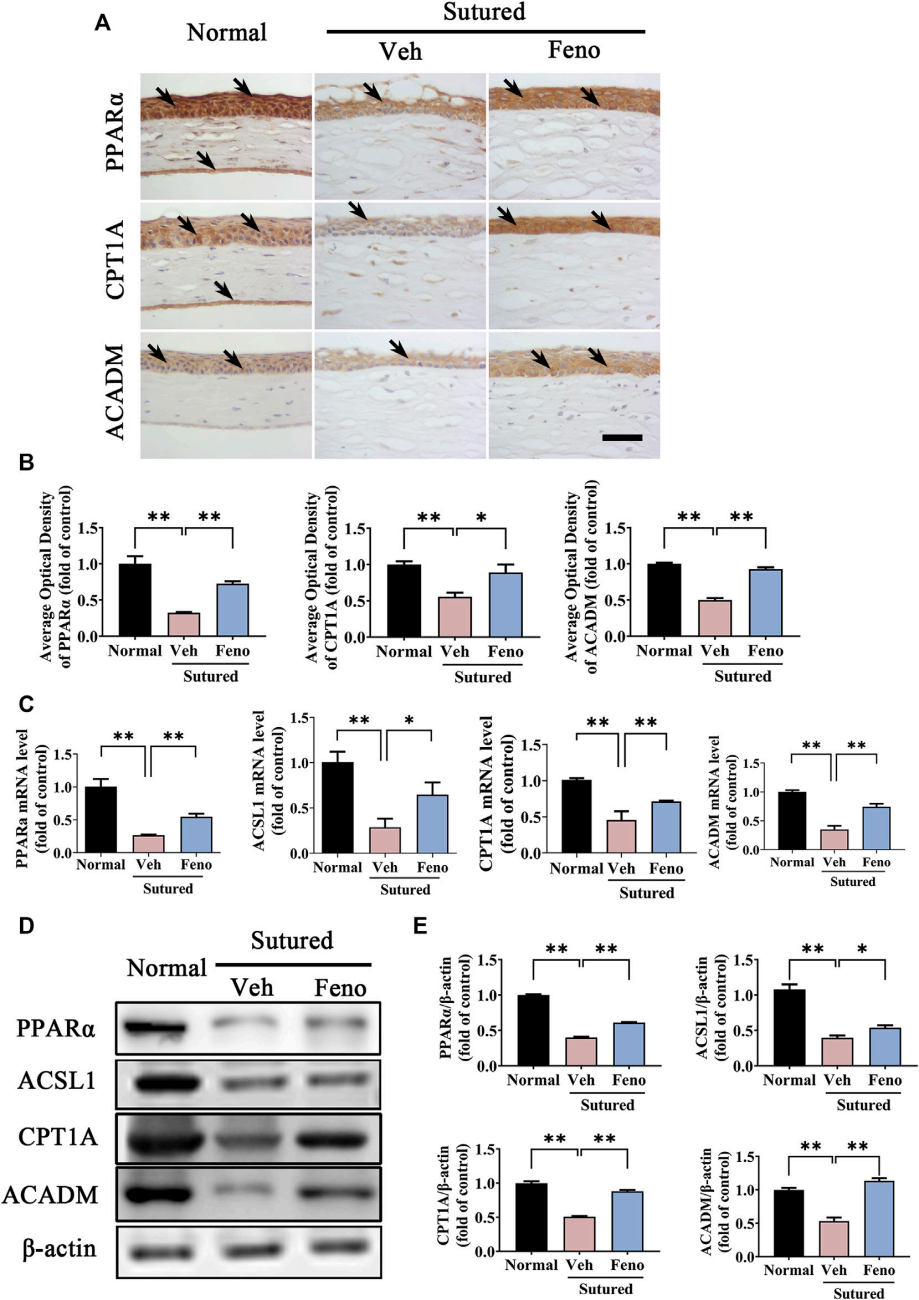
Fenofibrate upregulated the expression levels of lipid metabolism related enzymes in CNV. **(A,B)** Representative images of IHC staining (brown labeling, which indicated by black arrows) of cornea paraffin sections stained with antibody of PPARα, CPT1A and ACADM and the statistic analysis of the IHC staining between groups. Scale bar: 50 μm, n = 4 in each group. **(C)** Statistic analysis of qRT-PCR assay of expression of *Pparα*, *Ascl1*, *Cpt1a* and *Acadm* between groups, n = 3 in each group. **(D,E)** Images and analysis of western blot with antibody of PPARα, ASCL1, CPT1A and ACADM. The mice were divided into three groups as before, n = 4 in each group. ** denotes *p* < 0.01, * indicates *p* < 0.05, one-way analysis of variance (ANOVA). Data are presented as mean ± SEM.

To verify the role of lipid metabolism related enzymes in the pathogenesis of CNV, we further studied the changes of PPARα pathway and its downstream target after fenofibrate treatment in CNV. It was demonstrated that expression level of PPARα was downregulated in the vehicle group obviously, while fenofibrate markedly inhibited the downregulation of PPARα induced by suture in IHC staining (Figures 7A,B, *p* < 0.01), qRT-PCR detection (Figure 7C, *p* < 0.01) and western blot (Figures 7D,E, *p* < 0.01). We also detected the changes of the lipid metabolism related enzymes, including CPT1A, ACSL1 and ACADM. The results of IHC staining (Figures 7A,B, *p* < 0.05), qRT-PCR detection (Figure 7C, *p* < 0.05) and western blot (Figures 7D,E, *p* < 0.05) revealed that fenofibrate significantly reversed the decreasing of CPT1A, ACSL1 and ACADM induced by suture. All the results suggested that fenofibrate might play a protective role in CNV by regulating PPARα pathway and its downstream genes related with lipid metabolism.

## 4 Discussion

PPARα was demonstrated to regulate blood vessel growth in various diseases including cancer, atherosclerosis and ocular diseases (Wagner and Wagner, 2020; Dou et al., 2021). The decreased expression level of PPARα had been found to be associated with retinal inflammation and choroidal neovascularization (Abcouwer, 2013; Chen et al., 2013). In recent years, lots of studies showed that PPARα agonists exert therapeutic effects against ocular degenerative disorders with effects on anti-inflammatory, anticoagulant, anti-apoptosis, anti-oxidation and inhibition of neovascularization (Deng et al., 2017; He et al., 2020; Nakano et al., 2020; Nakano et al., 2020), while PPARα−/− mice with diabetes manifest more severe retinal acellular capillaries (Moran et al., 2014; Qiu et al., 2017). It was also demonstrated that PPARα plays a critical role in the regulation of inflammatory process and squamous metaplasia in ocular surface (He et al., 2020). In addition, PPARα agonists had therapeutic effects against corneal neovascularization induce by alkali burn or FGF2 (Panigrahy et al., 2008). The morphological characteristics of CNV were distinct in patients suffering from different factors in clinics. The strong inflammation induced by alkali injury to the mouse cornea leads to corneal epithelial defects, eyelid burns, corneal ulcers, scars and neovascularization (Cabalag et al., 2015; Nakano et al., 2020). Moreover, CNV model induced by suture in animal models displayed better practical advantages (Shi et al., 2010; Giacomini et al., 2014). Thus, we used the CNV model induced by suturation in our present study, which was a more dominant and specific model to investigate the pathogenesis and treatment of CNV. In our work, PPARα was mainly located on the corneal epithelium and stroma, and its expression was decreased in suture-induced CNV model, which was the same as demonstrated in other models (Wang et al., 2020). Here, we confirmed that fenofibrate exhibited the anti-neovascular effects in the corneal suture model through the regulation of PPARα.

PPARs are critical regulators of metabolism and PPARα activation lowers free fatty acids *via* upregulating the synthesis of proteins responsible for fatty acid β-oxidation and transport, which affects the formation of triglycerides and very low-density lipoprotein (VLDL) (Noonan et al., 2013). Experimental evidence indicated that PPARs may influence intracellular signaling pathways that lead to microvascular formation through suppressing lipotoxicity (Hiukka et al., 2010), regulating lipid metabolic products (Gong et al., 2016b) and fatty acid-binding proteins (FABP) (Yu et al., 2016). In our study, Lipi-Green staining and MDA measurement showed that lipid deposition and peroxidation were increased in the CNV cornea. In fact, lipid peroxidation began with the previous accumulation of lipid peroxides, while lipid peroxidation peaked at day 5, while the length of corneal neovascularization was gradually increased after suture with peak numbers by day 7. Lipid deposition, peroxidation and CNV were presented in sequential order. We concluded that the formation of CNV might be associated with the dysregulation of lipid metabolism and fenofibrate might protected cornea from suture-induced neovascularization. Lipi-Green staining suggested lipid deposition was decreased in the fenofibrate-treated group compared with the vehicle group. Meanwhile the MDA measurement showed more lipid peroxidation products and TUNEL staining revealed more apoptotic epithelial cells in suture-induced NV cornea. Nevertheless, fenofibrate treatment reversed these alterations after suture. It has been proposed that lipid metabolism by PPARα regulation was closely related with inflammation reaction in several diseases (Bougarne et al., 2018). Excessive inflammatory factors and pro-angiogenesis factors were believed to contribute to the formation of CNV (Sharif and Sharif, 2019). In our study, fenofibrate also played anti-inflammatory and anti-VEGF effects on suture mice. Therefore, lipid peroxidation products were accumulated in suture-induced NV cornea with subsequently cornea damage and inflammation, and fenofibrate might play anti-neovascularization effects *via* inhibiting the abnormal lipids metabolism. It has been reported that inhibition of ALOX5, a member of the lipoxygenase family catalyzing the oxygenation of arachidonic acid, exerted anti-angiogenic effect *in vitro* (Lim et al., 2019). In this study, fenofibrate significantly reversed the enhanced expression level of ALOX5 in suture-induced CNV model. It was indicated that lipid accumulation in CNV cornea might induce the expression of lipoxygenase and lipid peroxidation subsequently to cause corneal injury. Fenofibrate seemed to inhibit this process *via* inhibiting ALOX5 expression.

The corneal epithelial structural and functional integrity is vital for barrier function(Kim et al., 2014). Any changes occurred in the barrier function will affect corneal transparency, that leads to an increased risk of infections, corneal damage and corneal diseases. Fortunately, the presence of limbal stem cells (LSCs) guarantees corneal epithelium continuously renewal and wound healing, which play critical roles in protection of the ocular structures and function(Lobo et al., 2016). Corneal epithelium apoptosis is also an early response to corneal epithelial injury in cornea (Nowell et al., 2016). Corneal epithelial structural integrity was disrupted, with compromised epithelial barrier function after suture. Besides lipid peroxidation, increased apoptosis of the epithelial cells were also associated with the corneal epithelial injury repair on D1. Maintaining lipid metabolic enzymes homeostatic levels on epithelium has proven essential for preserving the integrity of the epithelial barrier. The corneal epithelium has important homeostatic functions in maintaining corneal transparency(Mocan and Irkec, 2015). The corneal epithelium serves as a barrier and helps maintain the corneal transparency, while the corneal endothelial cells form a barrier between the cornea and the aqueous humor, maintaining corneal transparency through its ionic pump function and barrier (Yamaguchi et al., 2020).

The lipid layer plays a key role in the stability of tear film on the ocular surface (Viitaja et al., 2021), and lipid metabolic enzymes in corneal epithelial are essential to maintain corneal transparency and integrity. In our previous study we found CPT1A, the rate-limiting enzyme of FAO, was downregulated in cornea of a dry eye model induced by sleep deprivation (Tang et al., 2018). It was reported that FAO is important for vessel sprouting in cancer and CPT1A might play critical roles in the endothelial cell proliferation and pathological angiogenesis (Harjes et al., 2016; Schlaepfer and Joshi, 2020). CPT1A was also overexpressed in many human tumors because of the uncontrolled proliferation of cancer cells (Iwamoto et al., 2018). Of note, inhibition of CPT1A by etomoxir or oxfenicine induced a dose-dependent increase of blood vessel leakage in mice (Patella et al., 2015). These results revealed the importance of lipid metabolism equilibrium between blood endothelial cells and surrounding cells, for example corneal epithelium or keratocyte. Normal aqueous humor has been shown to contain more than 400 lipid species(Jahn et al., 1983; Collao et al., 2022; Liu et al., 2022), and the corneal endothelial cells serves as a dynamic barrier that separates aqueous humor from corneal stroma (Hasan and Fischer, 2021). It has been widely reported that increased intake of ω-3 long-chain polyunsaturated fatty acids (LCPUFA) diets reduce choroidal neovascularization in mice (Gong et al., 2016a; Gong et al., 2022). Adiponectin was a polypeptide hormone secreted mainly by the adipose tissue with anti-inflammatory, anti-angiogenesis, anti-diabetic, anti-atherosclerotic and anti-tumoral effects (Baradaran-Rafii et al., 2021). Our previous studies shown that hyperlipidemia affectted tight junctions and pump function in the corneal endothelium (Bu et al., 2020). In our study, we focused our mechanism research on the key enzymes of FAO in lipid metabolism including ACSL1, CPT1A and ACADM, which were members of ACSL, CPT and ACAD families, respectively. We detected strong expression of ASCL1, CPT1A and ACADM in the normal cornea (especially in corneal epithelium and endothelium), indicating the physiological need of these factors for the lipid homeostasis maintenance of normal cornea. Interestingly, the mRNA and protein levels of all the three enzymes were reduced in NV cornea, and their reductions were reversed by fenofibrate. We concluded that the formation of CNV might be associated with the dysregulation of lipid metabolism and fenofibrate might protected cornea from suture-induced neovascularization through regulating the expression of ACSL1, CPT1A and ACADM in FAO.

In summary, in this study we found ACSL1, CPT1A and ACADM, which are key enzymes of lipid metabolism and downstream target genes of PPARα pathway, played important roles in the formation of CNV induced by suturation. PPARα agonist fenofibrate exerted the anti-neovascularization effect *via* reserving the levels of ASCL1, CPT1A and ACADM to ameliorate lipid deposition and oxidative damage in suture-induced CNV. Despite the contributions of the study, a few issues remain to be solved. A single dose of fenofibrate was used, and the dose ranges for optimal effects need to be defined. The cellular mechanism and signaling pathways mediating the role of fenofibrate on inflammation, angiogenesis and lipid metabolism need to be elucidated in more detail. In addition, CNV could not be completely inhibited by fenofibrate may connect with off-target side effects. It is necessary to determine whether the combination of fenofibrate and anti-VEGF have a synergistic effect, or to develop efficient and reliable PPARα agonists with chemical structures different from fenofibrate may avoid off-target side effects of fenofibrate.

## Data Availability

The original contributions presented in the study are included in the article/supplementary material, further inquiries can be directed to the corresponding authors.
